# Decreased NPC1L1 expression in the liver from Chinese female gallstone patients

**DOI:** 10.1186/1476-511X-9-17

**Published:** 2010-02-08

**Authors:** Wei Cui, Zhao-Yan Jiang, Qu Cai, Ru-Yuan Zhang, Wei-Ze Wu, Jian-Cheng Wang, Jian Fei, Sheng-Dao Zhang, Tian-Quan Han

**Affiliations:** 1 Department of Surgery, Ruijin Hospital, Shanghai Jiaotong University School of Medicine, Shanghai Institute of Digestive Surgery, 200025, Shanghai, PR China

## Abstract

**Background:**

Cholesterol gallstone disease is a very common disease in both industrialized and developing countries. Many studies have found that cholesterol gallstones are more common in women than men. The molecular mechanisms underlying the relationship between female gallstone disease and hepatic sterol transporters are still undergoing definition and have not been evaluated in humans.

**Aims:**

The aim of this study is to probe for underlying hepatic molecular defects associated with development of gallstones in female.

**Methods/Results:**

Fifty-seven nonobese, normolipidemic Chinese female gallstone patients (GS) were investigated with 12 age- and body mass index-matched female gallstone-free controls (GSF). The bile from the female GS had higher cholesterol saturation than that from the female GSF. The hepatic NPC1L1 mRNA levels were lower in female GS, correlated with SREBP2 mRNA. NPC1L1 downregulation was confirmed at protein levels. Consistently, immunohistochemistry showed decreased NPC1L1 expression in female GS.

**Conclusions:**

The decreased hepatic NPC1L1 levels in female GS might indicate a downregulated reabsorption of biliary cholesterol in the liver, which, in turn, leads to the cholesterol supersaturation of bile. Our data are consistent with the possibility that hepatic NPC1L1 may be mediated by SREBP2.

## Introduction

Cholesterol gallstone disease is common in both industrialized and developing countries [1,2]. Several clinical investigations have found an association between the increased incidence of cholesterol gallstones in China and a westernization of the traditional Chinese diet [2-4]. And because of the world-wide obesity epidemic with insulin resistance being part of the metabolic syndrome, the prevalence of cholesterol gallstones seems to be rising.

As found by epidemiological and clinical studies, women are twice as likely as men to form cholesterol gallstones at all ages, and estrogen could be an important risk factor for the formation of cholesterol gallstones. A large number of human and animal studies have proposed that estrogen increases the risk of developing cholesterol gallstones by increasing the hepatic secretion of biliary cholesterol.

Hypersecretion of biliary cholesterol and cholesterol supersaturation of the bile are considered to be the most important prerequisites for gallstone formation. Presently, the pathways for the biliary cholesterol secretion in the liver are incompletely understood. In a previous study, we observed that Chinese gallstone patients had an increased hepatic ABCG5/ABCG8 expression [5]. But study also found no relationship exists between biliary cholesterol excretion and hepatic ABCG5 and ABCG8 gene expression in human liver transplantation patients [6]. The existence of novel mechanisms other than ABCG5/ABCG8 in regulating biliary cholesterol secretion has been suggested based on several recent observations in mice [7,8].

Niemann-Pick C1-like 1(NPC1L1) has been recently identified as a critical protein for enterohepatic free cholesterol absorption [9-13]. NPC1L1 is widely expressed in many human tissues, with the highest expression in small intestine and in the liver [13,14]. Recent studies have identified NPC1L1 protein may play an important role in the hepatic secretion of cholesterol. Hepatic overexpression of NPC1L1 in mice significantly decreases biliary cholesterol concentration [9].

Compared with female gallstone-free controls (GSF), the metabolic abnormalities underlying the supersaturation of bile and the formation of cholesterol gallstones in female gallstone patients (GS) are not yet fully understood. In this study, we attempted to identify some of the molecular defects in hepatic cholesterol and bile acid metabolism involved in the pathogenesis of cholesterol gallstone disease in female. Our present results suggest that in Chinese female GS, supersaturation of bile is associated with a decreased expression of hepatic NPC1L1. We also observed that hepatic NPC1L1 may be mediated by SREBP2.

## Materials and methods

### Subjects

Fifty-seven Chinese female GS and 12 Chinese female GSF were included in this study. The female GS underwent laparoscopic cholecystectomy. Gallbladder size was evaluated by ultrasonography, and gallbladder function was assessed by analysis of total biliary lipids. Cholesterol gallstones were confirmed by visual inspection of the typical cut-surface of gallstones or, when necessary, by enzymatic cholesterol analysis. The female GSF included nine patients with right hepatic hemangioma and three with early carcinoma of head of pancreas, who also undergoing cholecystectomy. No gallstones were found in any of these controls after resection of the gallbladder, nor were cholesterol crystals found in bile by polarized light microscopy. None of the patients had any other disorders affecting hepatic, gastrointestinal, renal, and endocrine functions (i.e., either diabetes mellitus or signs of insulin resistance). None of the patients were subjected to any lipid-lowering treatment. Informed consent was obtained from all participants prior to enrollment in the study, including permission to collect a liver biopsy. The study protocol was approved by the ethics committees at the Ruijin Hospital, Medical School of Shanghai Jiaotong University.

### Procedure for sample collection

Patients were fasted overnight before surgery, which began at 9 AM. After opening the abdomen, or after the application of pneumoperitoneum, a wedge biopsy (~0.5-1.0 g) was taken from the right lobe of the liver, immediately snapfrozen in liquid nitrogen, and stored at -80°C. Criteria for a functioning gallbladder consisted of i) the presence of dark concentrated bile in the gallbladder and ii) no evidence of impacted stones in the neck of the cystic duct at operation. After clamping the cystic duct, bile from the gallbladder was obtained by aspiration. All of the operations were performed without any complications. Participation in the study did not result in prolonged hospitalization, and no serious adverse events were reported.

### Plasma and biliary lipid analyses

Plasma total cholesterol, triglycerides, HDL cholesterol, apolipoprotein A-I (apoA-I), and apoB were analyzed with an automated bioanalyzer (Roche Hitachi Modular P800) and corrected by dilution with the addition of EDTA (2%). LDL cholesterol in plasma was calculated according to Friedewald's equation. Gallbladder bile cholesterol, phospholipid and total bile salt levels were measured as described previously [15]. The cholesterol saturation index (CSI) was calculated using Carey's critical table [16].

### Relative mRNA quantification

Hepatic total RNA was extracted using Trizol reagent (Invitrogen, Carlsbad, CA) according to the manufacturer's protocol, and quality was evaluated by measuring the 260/280-nm absorbance ratio (≥1.8) and by electrophoresis. cDNA synthesis was performed with the High-Capacity cDNA Reverse Transcription Kit (Applied Biosystems, Foster City, CA, USA). Real-time quantitative PCR assays were performed in triplicate using SYBR-Green (MedProbe, Oslo, Norway). PCR primers (primer sequences are available on request) were designed using Primer Express 2.0 (Applied Biosystems, Foster City, CA, USA), all with sequences crossing exon-exon boundaries. Data were calculated by the ΔC_t _method, expressed in arbitrary units, and normalized to the signals obtained when the same cDNA was assayed for Cyclophilin A, selected as endogenous reference gene. The fold change for each mRNA quantity in the female GS material was expressed in relation to the obtained value for female GSF, the mean value of which arbitrarily was set at 1.

### Western blot analysis

Fifty micrograms of liver membranes from each patient sample was separated on a 10% SDS-PAGE gel and then transferred onto nitrocellulose membranes (Invitrogen). After blocking in 5% nonfat dry milk in PBST (PBS with 0.05% Tween-20), the nitrocellulose membranes were incubated overnight at 4°C with goat anti-NPC1L1 (1:200; Santa Cruz Biotechnology, Inc., Santa Cruz, CA, USA) in 5% nonfat milk powder in PBST. After washing with PBST, donkey anti-goat IgG antibody was added (1:2,000; Santa Cruz). The signals were detected using the SuperSignal chemiluminescence kit (Pierce Biotechnology, Inc., Rockford, IL) and a Fuji BAS 1800 analyzer (Fuji Photo Film Co.) and quantified by ImageJ 1.37 http://rsb.info.nih.gov/ij. After cleaning the membranes by stripping, they were further blotted with mouse anti-GAPDH (1:5,000; Kangchen Biotech, Shanghai, China) as a loading control. Data are expressed as arbitrary units and normalized to GAPDH expression.

### Immunohistochemical analysis

A single expert pathologist unaware of gene expression data evaluated all specimens. NPC1L1 intracellular and plasma membrane (PM) localization were detected by immunohistochemistry on paraffin-embedded tissue samples using the antibody (Rabbit Polyclonal anti-Niemann-Pick type C1 Like-1, Novus Biologicals, Inc., Littleton, CO, USA) in all subjects. Sections (5 μm thick) were obtained from formalin-fixed, paraffin-embedded specimens. Briefly, tissue samples were deparaffinized and hydrated, treated with citrate buffer (0.01 mol/l, pH 6) to unmask antigens, and treated with 3% H_2_O_2 _to block endogenous peroxidase. Samples were then incubated with the primary antibody at 1:50 dilution and washed. The reaction was performed using the EnVision Dako system (Dako, Milan, Italy) and developed by 3',3'-diaminobenzidine (DAB). Pre-immune rabbit serum, instead of primary antibody, was used as a negative control, since there was no signal. A positive reaction was indicated by a reddish-brown precipitate in the cytoplasm or PM.

Sections were viewed with a Nikon ECLIPSE E600 microscope (Nikon, Japan) using 10×, 20× and 40× objective lenses, and images were acquired with a SPOT INSIGHT™ digital colour camera, model 3.2.0 (Sterling Heights, MI). Quantification of immunoreactivity was performed on digitally captured colour images saved as tiff files and analysed using ImageJ 1.37 http://rsb.info.nih.gov/ij as described previously [17].

### Statistical Method

All data are expressed as means ± SD. Statistically significant differences between groups were assessed by the Student's t-test, Mann-Whitney U test, and correlations were performed by Pearson's test. Statistical analyses were performed using SPSS 11.0 for Windows (SPSS; Chicago, IL). Statistical significance was defined as a 2-tailed probability of less than .05.

## Results

### Clinic characteristics and plasma lipids

Demographic data for female GS and female GSF are shown in Table 1. No significant differences in age and body mass index were observed. No differences in plasma lipids between female GS and female GSF were present (Table 1).

**Table 1:** Age, body mass index, and plasma lipids of female GS and female GSF

Variable	Female GS (n = 57)	Female GSF (n = 12)
Age (years)	47.3 ± 14.2	48.6 ± 16.6
Body mass index (kg/m^2^)	22.8 ± 1.3	22.09 ± 2.2
Cholesterol (mmol/l)	4.18 ± 0.37	4.13 ± 0.46
Triglyceride (mmol/l)	1.68 ± 0.26	1.79 ± 0.17
HDL (mmol/l)	1.17 ± 0.06	1.18 ± 0.08
LDL (mmol/l)	2.23 ± 0.22	2.16 ± 0.25
Apolipoprotein A-I (g/l)	1.21 ± 0.06	1.19 ± 0.09
Apolipoprotein B (g/l)	0.71 ± 0.05	0.72 ± 0.06

GS, gallstone patients; GSF, gallstone-free controls.

### Biliary lipid composition

As shown in Table 2, in the samples analyzed, a significantly greater molar percentage of cholesterol was present in the bile of the female GS compared with the female GSF, as well as a significantly greater cholesterol saturation index (female GS vs. female GSF, 1.09 ± 0.07 vs. 0.70 ± 0.05; P < 0.01). Neither the total bile acids nor the phospholipids in bile differed between the groups (Table 2). No differences were found in total biliary lipids (female GS vs. female GSF, 12.78 ± 1.57 vs. 13.32 ± 1.54 g/dl; P = NS), possibly because we ensured that all of the female GS had normal gallbladder function.

**Table 2:** Biliary lipid composition in female GS and female GSF

Variable	Female GS (n = 57)	Female GSF (n = 12)
Cholesterol (mmol/L)	16.76 ± 1.79	11.32 ± 1.06
Phospholipids (mmol/L)	44.06 ± 4.37	52.39 ± 4.58
Bile acids (mmol/L)	131.45 ± 14.56	173.58 ± 15.89
Cholesterol (mol%)	7.96 ± 0.46**	4.78 ± 0.38
Phospholipids (mol%)	22.01 ± 1.07	21.97 ± 0.96
Bile acids (mol%)	71.92 ± 1.23	74.76 ± 1.43
Total biliary lipids (g/dl)	12.78 ± 1.57	13.32 ± 1.54
CSI	1.09 ± 0.07**	0.70 ± 0.05

GS, gallstone patients; GSF, gallstone-free controls; CSI, cholesterol saturation index; ** female GS vs. female GSF, P < 0.01.

### Hepatic gene mRNA levels

**Figure 1:**
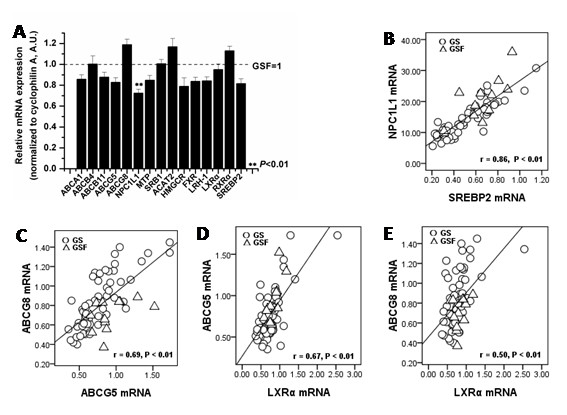
**Quantitative mRNA expression levels in genes from the liver in female gallstone patients (GS) and female gallstone-free patients (GSF)**. A: Relative gene expression between female GS (n = 57) and female GSF (n = 12). The dotted line at value 1 represents the mean gene expression level in female GSF, which was arbitrarily set to 1 (A.U.); the black bars represent the gene expression levels in female GS (means ± SD). B: Correlation between hepatic NPC1L1 and SREBP2 mRNA levels (n = 69). C: Correlation between hepatic ABCG5 and ABCG8 mRNA levels (n = 69). D: Correlation between hepatic LXRα and ABCG5 mRNA levels (n = 69). E: Correlation between hepatic LXRα and ABCG8 mRNA levels (n = 69).

As shown in Fig. 1A, the mRNA levels for the hepatic NPC1L1 were lower in the female GS compared with the female GSF (P < 0.01). Interestingly, the expression of ABCG5, ABCG8 and LXRa, measured as mRNA abundance, did not differ significantly between female GS and female GSF, as was also the case for 11 other genes involved in various aspects of the regulation of hepatic lipid metabolism (Fig. 1A).

A strong positive correlation between hepatic NPC1L1 and SREBP2 mRNA expression was present (r = 0.86, P < 0.01) (Fig. 1B), suggesting that SREBP2 may be an important regulator of the hepatic expression of NPC1L1 in humans. NPC1L1 expression also correlated negatively with the biliary cholesterol molar percentage (r = -0.73, P < 0.01) and the CSI (r = -0.76, P < 0.01) (data not shown).

Although, there were no differences in hepatic ABCG5, ABCG8 and LXRa mRNA expression between female GS and female GSF in our study, we found that ABCG5 and ABCG8 mRNA levels correlated very well (r = 0.69, P < 0.01) (Fig. 1C), confirming their likely coexpression in human liver in vivo, as we previously have observed in liver tissue [5]. Because LXRa are known to regulate ABCG5/ABCG8, these genes were used as controls of our model; also, we tested the correlation between LXRa and ABCG5/ABCG8. In agreement with previous studies, we found that LXRa correlated with both ABCG5 (r = 0.67, P < 0.01) (Fig. 1D) and ABCG8 (r = 0.50, P < 0.01) (Fig. 1E) mRNA levels.

### NPC1L1 protein expression correlate with the mRNA levels

**Figure 2:**
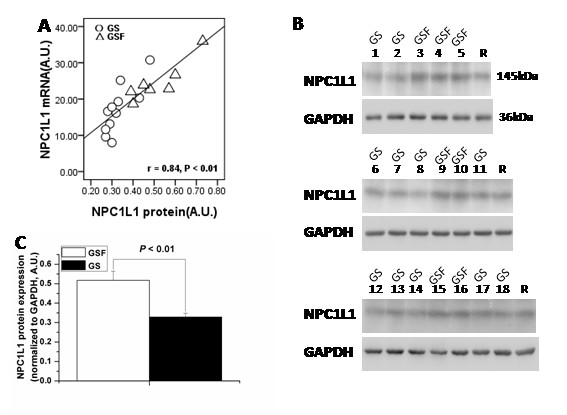
**Hepatic expression of NPC1L1 protein in female gallstone patients (GS) and female gallstone-free patients (GSF)**. A: Correlation between NPC1L1 mRNA and protein levels in 18 female subjects (*P *< 0.01). AU, arbitrary units. B: NPC1L1 protein expression in female patients with gallstone disease (n = 11), and without gallstone disease (n = 7). GAPDH is shown as a control. R represents a human liver membrane sample that was used as a reference for each gel. C: NPC1L1 protein level was lower in female GS than in female GSF. Data show means ± SD of the value obtained from the quantitation of the blots shown in 2B. A.U., arbitrary units.

To determine whether decreased NPC1L1 mRNA levels translated into decreased activity, we analyzed the relationship between normalized mRNA and protein levels. We observed a significant correlation between NPC1L1 mRNA and protein levels (n = 18, r = 0.84, P < 0.01) (Fig. 2A).

NPC1L1 displayed lower levels of mRNA in female GS compared with female GSF (P < 0.01) (Fig. 1A). This was paralleled by decrease of the NPC1L1 protein (P < 0.01), NPC1L1 protein levels were lower in female patients with gallstone disease than in those without gallstone disease (Fig. 2B and Fig. 2C).

### NPC1L1 location and expression in hepatocytes by immunohistochemistry

**Figure 3:**
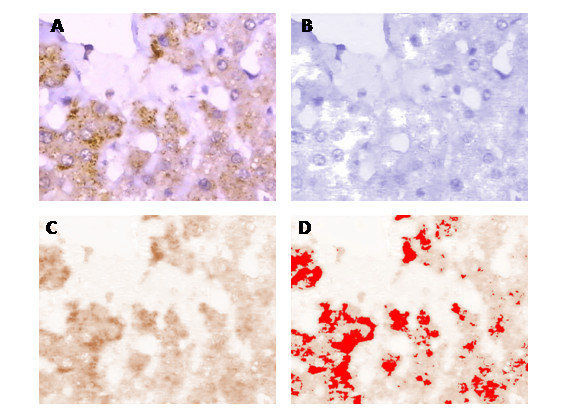
**Quantitative immunohistochemistry to detect NPC1L1 from a specimen from a female gallstone-free control using ImageJ**. After stripping the original digital image of non-white background (A), colour deconvolution is used to separate the haematoxylin component (B) from the 3',3'-diaminobenzidine (DAB) component (C). The minimum and maximum mean optical density values of eight positively stained cells selected from the DAB analysis are used to set the threshold manually (D) for sample optical density measurement. original magnification 40×.

After subtracting the non-white background colour, the haematoxylin component was separated from the DAB component using colour deconvolution as described [18] (Fig. 3A-C). For each specimen, the lowest and the highest mean optical density (MOD) values measured from eight manually identified positively stained cells selected from the DAB component (with visual comparison with the original image) was used to set the threshold manually for whole sample staining density quantification (Fig. 3D). Comparison of panels A and D indicates that NPC1L1 is expressed in both the cytoplasm and PM of the hepatocytes.

**Figure 4:**
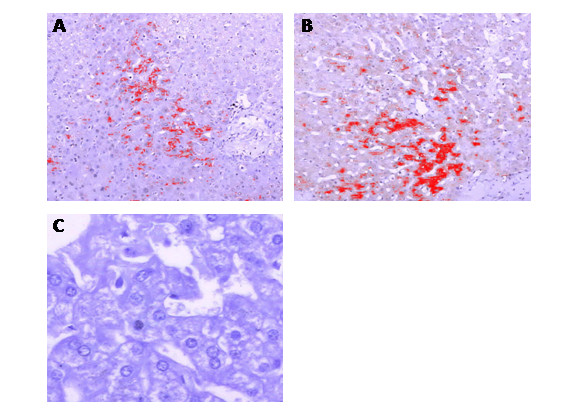
**Presence of hepatic NPC1L1 in female gallstone patients (GS) and female gallstone-free patients (GSF)**. Tissues were immunohistochemically stained for NPC1L1 (A, B). Hepatic specimens shown are from female GS (A) and female GSF (B). Panel C shows results in the absence of primary antibody for NPC1L1. Panels A, B are superimposed with optical density results (red colour) derived from ImageJ. All samples were counterstained with haematoxylin; original magnification 10× (A, B) and 40× (C).

Quantitative immunohistochemistry (Fig. 4A, B) was performed to compare hepatic NPC1L1 expression between female GS and female GSF. The mean area of the evaluated specimens, measured in square pixels, did not vary significantly between the two groups (female GS, 1 112 900 ± 171 037; female GSF, 1 156 300 ± 159 485 (P > 0.05)). There was a trend towards a lower mean percentage area showing NPC1L1 positivity in the female GS samples (5.4 ± 2.1%) compared with female GSF samples (12.9 ± 4.2%) (Fig. 4A, B), and the difference was statistically significant (P < 0.01). The NPC1L1 immunohistochemical MOD differed significantly (P < 0.05) between the female GS (0.33 ± 0.05) and female GSF (0.54 ± 0.07).

## Discussion

In order to identify the molecular defects in the pathogenesis of cholesterol gallstone disease, many previous studies have been carried out in animal models and have not been evaluated in humans. The present study is one of the first approaches to utilize liver biopsies to evaluate differences in gene expression between female patients with and without cholesterol gallstone disease. Our results confirm and extend findings in animal models, indicating that decreased hepatic NPC1L1 activity may play a role in the pathogenesis of gallstone disease in female.

Although cholesterol gallstone disease is a multifactorial disease influenced by a complex interaction of genetic and environmental factors [19], hepatic hypersecretion of biliary cholesterol is the primary defect in the formation of cholesterol gallstones. Recent studies on molecular transporters involved in biliary lipid secretion suggest that the hepatic secretion efficiency of biliary cholesterol could be regulated by multiple genes at the hepatocyte level, included the ABCG5/ABCG8-dependent and independent pathways [20]. Moreover, recent progress in understanding the molecular basis of hepatic sterol transporters has strongly suggested an ABCG5/ABCG8-independent pathway for hepatic secretion of biliary cholesterol and its role in the formation of cholesterol gallstones [20].

The NPC1L1 protein shares 42% identity and 51% similarity with NPC1 [12]. NPC1L1 is abundantly expressed in both the small intestine and liver of humans and localizes at the canalicular membrane of hepatocytes [9,10,21]. The canalicular expression of NPC1L1 in the liver resulted in a drastic reduction in biliary cholesterol concentration in 2 independent transgenic mouse lines [9], indicating that liver NPC1L1 may mediate biliary cholesterol reabsorption.

However, little is known about the real role of hepatic NPC1L1 expression in gallstone disease in humans. In another study made by us, no difference was found for the hepatic NPC1L1 mRNA levels between Chinese male GS (n = 30) and male GSF (n = 15) (data not shown). Interestingly, in our present study, decreased NPC1L1 mRNA and protein levels have been observed in the liver in female patients with gallstone disease, also suggesting possible similarity between transcriptional and translational actions in this hepatic cholesterol transporter. Further more, immunohistochemical evaluation supports this conclusion by showing decreased NPC1L1 hepatocellular staining in female patients with gallstone disease.

We did not observe any differences in plasma lipids between female GS and female GSF, an observation consistent with our previous studies in Chinese gallstone patients [5,22]. In our study, we found that a significantly greater molar percentage of biliary cholesterol and an increased CSI occurred in Chinese female gallstone patients. Moreover, we found hepatic NPC1L1 expression level also correlated negatively with the biliary cholesterol molar percentage and the CSI, which suggest the relationship between the expression level of hepatic NPC1L1 and biliary cholesterol secretion in humans. The functional impact of lower hepatic NPC1L1 expression in our female GS patients is difficult to estimate directly, but it could signify decreased biliary cholesterol reabsorption in the liver. This is also the first study to characterize such function for NPC1L1 in humans.

Some previous studies have shown that NPC1L1 localizes on the PM [10,23] or in intracellular compartments [13,14]. Recent study found that NPC1L1 recycles between endocytic recycling compartment (ERC) and PM, and the subcellular localization of NPC1L1 protein is regulated by cholesterol [24]. Cholesterol-depletion induces the translocation of NPC1L1 from ERC to PM, the NH2-terminus of NPC1L1 with a predicted signal peptide faces the extracellular matrix and the COOH-terminus projects to the cytosol [25], whereas replenishment of cholesterol results in the transportation of NPC1L1 from PM to ERC [24]. ERC is a component of the endocytic recycling system [26], NPC1L1 is in the ERC under normal condition [24], accordingly, a large NPC1L1 pool exists at the cell interior [27], consistent with our findings on NPC1L1 hepatocellular staining by immunohistochemistry.

Sterol regulatory element binding proteins (SREBPs) are transcription factors that are crucial regulators of cholesterol homeostasis [28]. Previous study found that in SREBP-1a, -1c, and -2 isoforms of this family of transcription factors, SREBP2 is the major regulator of the human NPC1L1 promoter [29]. And SREBP2 seems to be an important regulator of both the intestinal and the hepatic NPC1L1 expression in humans [29,30]. Our present study also showed a strong positive correlation between NPC1L1 and SREBP2 mRNA expression in liver from Chinese female patients with and without gallstone, which suggests that SREBP2 may be an important regulator of the hepatic expression of NPC1L1 in humans, in agreement with our previous report [30].

ABCG5 and ABCG8 appear to function as a heterodimer for the secretion of cholesterol into the bile canaliculus [31]. ABCG5/ABCG8 are direct target genes of the oxysterol-activated nuclear receptor LXRa [32,33]. In our previous study, we found higher levels of hepatic ABCG5/ABCG8 and LXRa mRNA in gallstone patients [5], which could cause higher amounts of cholesterol to be delivered into the bile. Interestingly, in the present study, no differences were observed between female patients and controls for the hepatic ABCG5/ABCG8 and LXRa mRNA expression, suggesting that gallstone disease is a multifactorial disease that is influenced by multiple genes. And we thought besides gender, the selection of the types of gallstone-free patients is an important reason for this discrepancy. In our present study and in the previous study, the gallstone-free controls were quite different. In our previous study, the majority of the patients enrolled in the gallstone-free controls were patients with gallbladder polyps. In the present study, the patients enrolled in the gallstone-free controls underwent surgical procedures because of right hepatic hemangioma or early carcinoma of head of pancreas, they all had perfectly normal gallbladder function.

However, in our female GS and GSF, we also showed a strong positive relationship between ABCG5 and ABCG8 mRNA expression levels, and ABCG5/ABCG8 also correlated with LXRa expression, consistent with these possibilities that in human liver, ABCG5 and ABCG8 are partners in the generation of a functional transporter, and ABCG5/ABCG8 are transcriptionally regulated by LXRa. Moreover, our female gallstone patients did not show any differences in the hepatic mRNA expressions of ABCB4, ABCB11, FXR and other genes participating in the hepatic lipid metabolism, in agreement with our previous study [5].

Our observation suggests that the hepatic NPC1L1 may be an important, selective target for the treatment of cholesterol gallstones in female. However, further studies are required to elucidate why decreased NPC1L1 expression occurred in female patients with gallstones, but not male gallstone patients (data not show). Furthermore, the relatively small sample size and the lack of prospective evaluation suggest caution in interpreting our result. And we consider that any conclusion regarding the exact role of NPC1L1 in cholesterol metabolism and transport should be appropriately interpreted and will also require further characterization.

In conclusion, in our study of normolipidemic, nonobese Chinese female gallstone patients, the supersaturation of the bile with cholesterol was associated with a decreased expression of hepatic NPC1L1, which was possibly mediated by hepatic SREBP2. The current result also suggests that to regulate hepatic NPC1L1 expression could represent a novel strategy for the prevention of cholesterol gallstones in female.

## List of Abbreviations Used

ACAT: acyl-coenzyme A: cholesteryl acyltransferase; ABC: ATP binding cassette; apoA-I: apolipoprotein A-I; CSI: cholesterol saturation index; ERC: endocytic recycling compartment; F×R: farnesoid × receptor; GS: gallstone patients; GSF: gallstone-free controls; HMGCR: 3-hydroxy-3-methylglutaryl coenzyme A reductase; LRH-1: liver receptor homologus-1; L×Ra: liver × receptor a; MOD: mean optical density; MTP: microsomal triglyceride transfer protein; NPC1L1: Niemann-Pick C1-like 1; PM: plasma membrane; R×R: retinoid × receptor; SRBI: scavenger receptor B type I; SREBP: sterol regulatory element binding protein.

## Competing interests

The authors declare that they have no competing interests.

## Authors' contributions

TQH obtained grant funds for project, designed study, supervised all study recruitment, data/specimen analysis, statistical analysis and manuscript preparation. WC was the lead author, participated in the design of the study, performed data collection, data/specimen analysis, statistical analysis and manuscript preparation. ZYJ, QC, RYZ, WZW, JCW, JF and SDZ were co-authors, oversaw all aspects of study including recruitment, data/specimen analysis, and manuscript preparation. All authors have read and approved the final manuscript.
